# Recent Developments in Layered Double Hydroxides as Anticorrosion Coatings

**DOI:** 10.3390/ma18153488

**Published:** 2025-07-25

**Authors:** Alessandra Varone, Riccardo Narducci, Alessandra Palombi, Subhan Rasulzade, Roberto Montanari, Maria Richetta

**Affiliations:** Department of Industrial Engineering, University of Rome Tor Vergata, Via del Politecnico 1, 00133 Rome, Italy; alessandra.varone@uniroma2.it (A.V.); riccardo.narducci@uniroma2.it (R.N.); alessandra.palombi@uniroma2.it (A.P.); subhan.rasulzade01@gmail.com (S.R.); roberto.montanari@uniroma2.it (R.M.)

**Keywords:** metals, corrosion, anticorrosion coatings, LDHs

## Abstract

To date, one of the main problems associated with the engineering application of metallic materials is corrosion protection. To increase their durability and reduce damage, a variety of protection methods have been studied and applied. In recent decades, coating techniques have become increasingly important. Among these coatings, Layered Double Hydroxides (LDHs) have shown unique properties, such as ion exchange, high adhesion, and hydrophobicity, particularly useful for biomedical applications. In this review, after a detailed exposition of the LDHs’ synthesis processes, the most recent corrosion protection methods are illustrated. Intercalation of corrosion inhibitors and release kinetics of intercalates are presented. Although this work is mainly focused on laboratory-scale investigations and fundamental research, the problems inherent to large-scale industrial manufacturing and application are outlined and briefly discussed.

## 1. Introduction

Corrosion is a widespread phenomenon that generates huge economic losses globally, with costs estimated between 1% and 5% of GDP in several countries [1,2]. It consists of an irreversible process of degradation of the surfaces of metals and alloys due to interaction with chemical agents that promote their transformation into thermodynamically and kinetically more stable forms [3,4]. This degradation occurs in the presence of specific conditions, which trigger reactions that compromise the functional properties and durability of materials used in industrial infrastructure [5,6,7]. They can be internal (composition, structure, defects, etc.) or external (temperature, pressure, pH, flow rate, environmental factors, etc.).

Corrosive processes, which are electrochemical in nature [8], are based on redox reactions that occur at the material surface and depend on the type of material, the corrosive environment, and the presence of additional chemical species, resulting in phenomena such as surface oxidation, pitting, cracking, and other localised damage. Such processes increase the risk of structural failure, malfunctioning, and operational safety problems. A detailed understanding of these mechanisms is crucial for corrosion prevention and control, to preserve the integrity of materials, extend their life, and optimise their performance [9].

**Figure 1:**
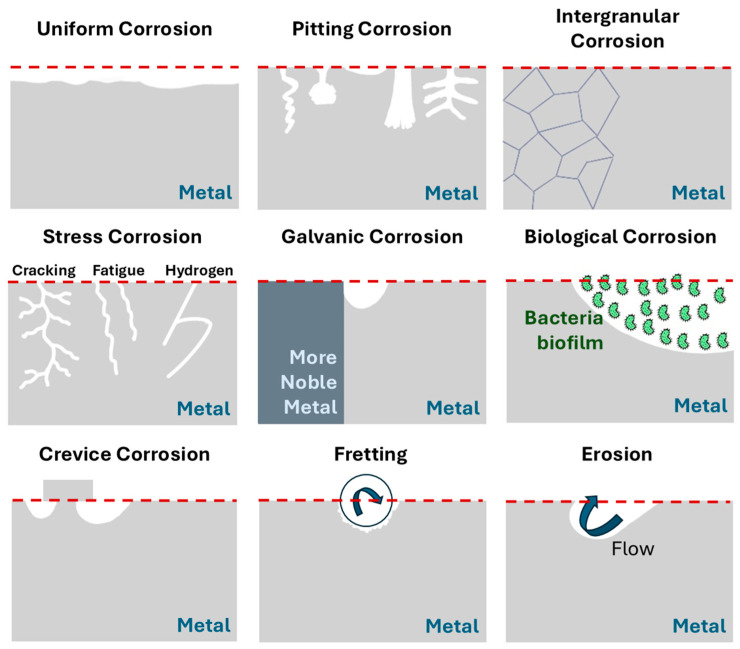
Schematic representation of corrosion forms.

Electrolytes facilitate the development of anodic and cathodic reactions and the transfer of electrons and ions, closing the electrochemical circuit necessary for corrosion [10]. The position of the anodic and cathodic “electrodes” can vary dynamically on the metal surface, facilitating the evolution of corrosion [11,12]. As shown in Figure 1, corrosion can manifest in different forms: uniform attack, localised attack, grain boundary attack, erosion, cavitation, fretting, biological attack, etc.

**Figure 2:**
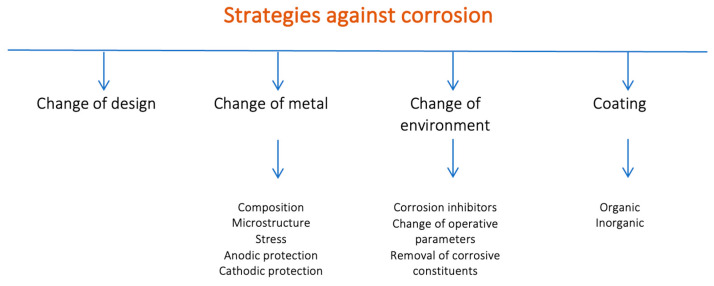
Anti-corrosion principal strategies.

Strategies against corrosion are substantially based on four approaches (see Figure 2) involving (i) the change of component design, (ii) the change of metal, (iii) the change of environmental conditions, and (iv) the use of coatings.

Traditional corrosion protection techniques, despite some limitations, have been used for decades to mitigate degradation induced by wear, UV radiation, and chemical attack. These methods require periodic maintenance, resulting in high costs and operational complexities for the industry. For example, it is estimated that the global annual cost of corrosion amounts to approximately $2.5 trillion. In specific sectors, such as oil and gas, corrosion-related expenses—including prevention, maintenance, and failure—can represent up to 20–30% of the total operating costs. Such figures highlight the economic urgency of developing more efficient and durable protection systems.

Among the most popular strategies is the use of sacrificial anodes, which are designed to oxidise preferentially to the metal substrate [13,14]. However, such approaches often show little flexibility, reduced effectiveness, and limited adaptability to the needs of modern industrial contexts. With the introduction of innovative materials and exposure to more aggressive environmental conditions, the development of advanced corrosion protection technologies is imperative [15,16].

Corrosion control techniques include processes such as galvanising, painting, dealloying, and the use of inhibitors [17], with the aim of reducing the corrosion rate to acceptable levels to ensure the expected life cycle of the materials. Some strategies aim at the complete elimination of corrosion.

In recent years, protective coatings have become increasingly important due to their ability to form effective physical barriers that prevent aggressive species from entering the metal interface [18,19]. In some cases, in addition to protecting against corrosion, such coatings improve the tribological properties of the substrate, reducing wear, friction, and surface damage.

In response to environmental and safety regulations, the use of toxic substances (Volatile Organic Compounds (VOCs), hazardous air pollutants, and hexavalent chromium (Cr(VI)) has been severely restricted. As a result, research has focused on the development of environmentally friendly and pollutant-free coatings in line with environmental sustainability and human safety requirements.

Industrial coatings mainly fall into two categories: inorganic and organic. Inorganic coatings, comprising ceramic and metallic materials (or alloys), offer significant improvements in terms of surface hardness, wear resistance, and corrosion protection [20]. These coatings can be applied through techniques such as thermal metallisation and electrodeposition. Among sacrificial anticorrosion coatings, zinc-rich primers are the most popular and effective solution, providing cathodic protection to ferrous substrates by forming stable corrosion products that seal any defects in the protective film, slowing down water absorption [21]. Other commonly used materials include alloys of titanium, nickel, magnesium, and silicon carbide, which are suitable for advanced sectors such as biomedicine, electronics, transport, and aerospace [22,23,24].

Organic coatings are widely used due to their versatility, ease of application, cost-effectiveness, and ability to provide high aesthetic performance [25]. They act as effective physical barriers against the penetration of corrosive agents into metal substrates [26]. However, the rapid deposition of such coatings generates numerous intrinsic micropores that can be trigger sites for defects, compromising durability and long-term corrosion resistance.

Numerous studies have focused on enhancing the corrosion resistance of organic coatings, with particular emphasis on the incorporation of fillers with lamellar morphology into the polymer matrix. Popular fillers include mica and glass flakes, which, when properly dispersed and oriented, generate tortuous pathways that significantly improve the physical barrier properties of the coating [27,28]. However, most of these fillers are micrometric in size and, in practical applications, a high content is required to ensure effective anti-corrosive performance. This requirement leads to difficulties in uniform dispersion and may compromise the inherent mechanical and chemical properties of the coating, reducing its overall effectiveness [29,30].

With the advancement of micro- and nanotechnologies, two-dimensional (2D) nanomaterials have taken centre stage as functional fillers for anti-corrosion coatings. Materials such as graphene, hexagonal boronitride (h-BN), and molybdenum disulphide (MoS_2_) offer multiple advantages: (a) enhancement of film density by effectively filling defects and reducing porosity [31]; (b) formation of continuous, dense physical barriers that hinder the diffusion of corrosive species [32,33]; (c) increased mechanical properties such as hardness, adhesion, and flexibility, resulting in increased durability under mechanical and environmental stress [30,33].

**Figure 3:**
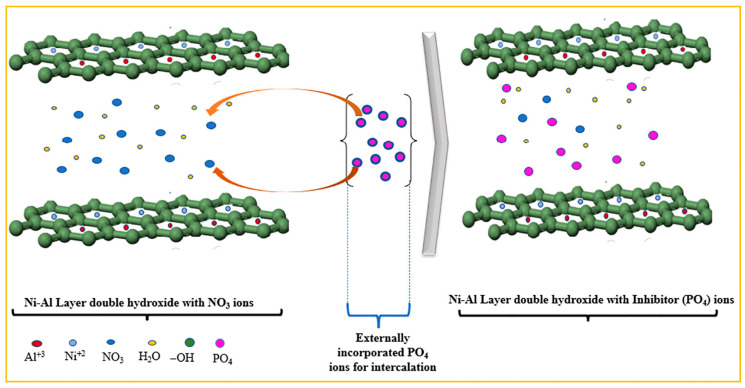
Intercalation of corrosion inhibitor (PO_4_) in Ni-Al-NO_3_ gallery. Replacing nitrate ions through the ion exchange method. Reproduced with permission from Surface & Coating Technology, published by Elsevier, 2024 [34].

Among these materials, Layered Double Hydroxides (LDHs) are distinguished by their unique lamellar structure and multifunctionality. LDHs offer a pronounced labyrinth effect that extends diffusion pathways, significantly slowing down ion transport. At the same time, their exceptional ion-exchange capacity allows efficient sequestration of aggressive ions, particularly chlorides, which are primarily responsible for localised corrosion in metals such as steel and aluminium alloys. This mechanism not only traps chloride ions but also facilitates the controlled release of corrosion inhibitors embedded in the interlamellar layers of LDHs, enabling active corrosion protection through a self-repair mechanism (cfr. Figure 3) [34,35].

In practical applications, it is well known that protective paint coatings, based on polymer matrices such as epoxy, polyurethane, acrylic, or alkyd resins, represent the most widely adopted approach for corrosion protection. These systems primarily function as passive barriers, limiting the ingress of water, oxygen, and chloride ions to the metal surface. However, they often suffer from intrinsic limitations, such as micropore formation during curing, degradation under UV or mechanical stress, and lack of active corrosion protection mechanisms.

In contrast, LDH-based coatings provide a dual-action mechanism:

- Passive protection, by enhancing the coating’s barrier properties through the tortuosity effect of the lamellar LDH structure, which slows the diffusion of aggressive species [36,37].
- Active protection, through the controlled release of corrosion inhibitors (e.g., molybdate, benzoate) intercalated in the LDH layers. These inhibitors are released in response to environmental triggers such as pH shifts or the presence of chloride ions, providing a self-healing function to the coating [36,38].

Furthermore, LDHs can be incorporated into polymeric matrices as nanofillers or directly synthesized onto metal surfaces. This versatility supports the development of smart and multifunctional anticorrosive systems, addressing the demands of advanced industrial sectors while aligning with environmental safety regulations [37].

Recent studies have demonstrated that LDHs can be successfully integrated into traditional anticorrosion paint formulations, particularly epoxy- and polyurethane-based systems, improving coating compactness, adhesion, and long-term corrosion resistance [36,37,38]. Their ion-exchange capacity and pH-responsiveness enable the formation of coatings capable of releasing inhibitors on demand, ensuring active mitigation of corrosion even in the presence of coating defects or microcracks. This approach enhances durability and offers a sustainable, chromium-free alternative to conventional systems.

As a further example, LDH-based nanofillers have been successfully incorporated into epoxy and polyurethane coatings for the protection of steel and aluminium alloys in marine and infrastructure environments, demonstrating prolonged service life in chloride-rich environments such as seawater and de-icing salts [39,40].

Within this context, recent studies have begun to explore the important aspects of the LDH lifecycle, addressing sustainability claims: (i) Huang et al. [41] reported LDH–carbon composites capable of >93% performance retention over five adsorption–desorption cycles, highlighting their potential for regeneration in wastewater applications; (ii) Jiang et al. [42] reviewed LDHs synthesized from industrial wastes such as red mud and fly ash, demonstrating pathways for circular economy integration; (iii) Mahgoub et al. [43] demonstrated successful recovery and reuse of Zn–Al LDHs post-adsorption of levofloxacin, confirming their recyclability as safe nanomaterials; (iv) Jing et al. [44] developed magnetic CaFe-LDHs recoverable via magnetic separation after cadmium immobilization in soil remediation, explicitly addressing end-of-life recovery strategies. These findings underscore both the promise and the limitations: while LDHs show good recyclability and lower environmental impact in controlled settings, comprehensive analyses of their lifecycle—including degradation, ecological fate, and large-scale end-of-life management—are still lacking.

However, despite these promising laboratory-scale results and sustainability-oriented developments, industrial case studies demonstrating long-term performance of LDH-based coatings remain scarce. Applications in critical sectors such as marine infrastructure, aerospace, and transportation often lack real-world validation through extended field testing. The absence of systematic performance data under operational conditions—including cyclic loading, UV exposure, and salt fog environments—limits the current understanding of LDHs’ reliability and durability in service. Closing this gap through field trials and industrial pilot studies is essential to establish the feasibility and robustness of LDHs in commercial corrosion protection applications [45,46].

**Figure 4:**
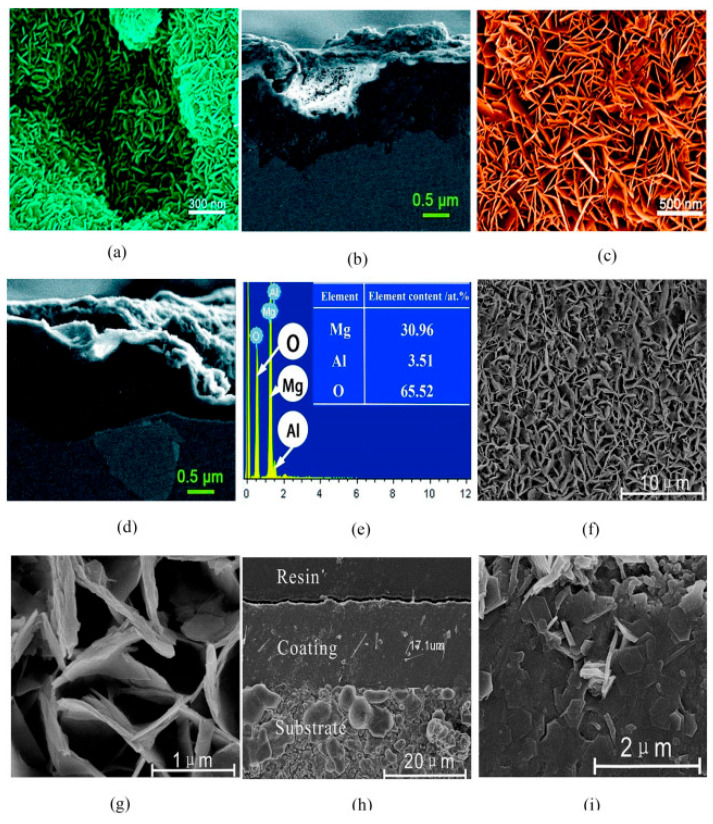
MgAl LDH physical protective film by the hydrothermal conversion of anodic layer on AZ31 Mg alloy: (**a**) SEM images of anodic oxidation layer, (**b**) Cross-sectional SEM images of anodic oxidation layer, (**c**) SEM images of MgAl LDH layer, (**d**) Cross-sectional SEM images of MgAl LDH layer, (**e**) EDS mapping of MgAl LDH layer, (**f**,**g**) SEM images of MoO_4_^2−^ intercalated MgAl LDH at different magnification, (**h**,**i**) Cross-sectional SEM images of MoO_4_^2−^ intercalated MgAl LDH at different magnification. Creative Commons CC-BY license [49].

Parallel to their use as nanofillers, LDHs can be also directly grown on the metal surface, as illustrated in Figure 4. The multifunctional nature of these 2D nanomaterials aligns with the growing demand for coatings that combine passive corrosion protection and active mitigation strategies, contributing to the development of next-generation smart coatings for harsh operating environments.

In addition to binary, there are also ternary LDHs consisting of three types of metal cations. In recent years, ternary LDHs such as ZnMgAl have been applied in the field of corrosion protection [47,48]. Jing et al. [49] reported that ZnMgAl LDH films exhibit excellent corrosion-inhibiting properties on aluminium foils. Compared to binary LDHs, the ternary ones allow a more precise control of the properties by modulating the types and proportions of the constituent metals. However, the synthesis conditions are more stringent and the associated costs higher [48].

**Figure 5:**
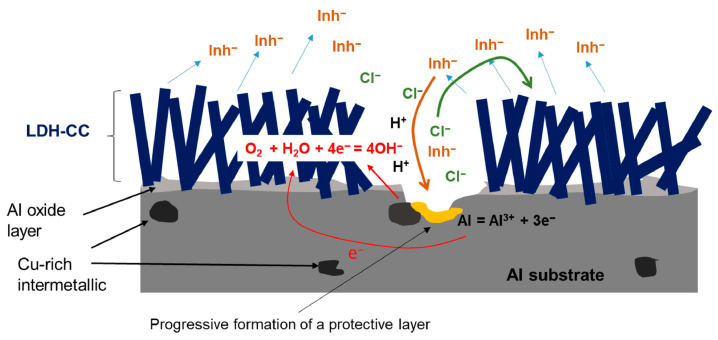
Scheme of the corrosion protection mechanism of LDH-CC prepared on an Al substrate. Reproduced with permission from Applied Materials Today, published by Elsevier, 2020. Creative Commons CC-BY license [51].

It thus emerges that LDHs are playing an increasingly important role in corrosion protection coatings, as evidenced by numerous reviews analysing the anticorrosive properties of LDH-reinforced organic coatings, focusing mainly on preparation strategies and applications of LDHs in the form of powders and films for corrosion protection [48]. Tabish et al. [50] highlighted the state-of-the-art nature of LDHs as nanocontainers for various inhibitors, capable of providing long-term corrosion protection. Furthermore, Bouali et al. [51] described how LDHs could act both as functional conversion coatings (CC) and as nanofillers, providing corrosion protection for aluminium alloys (cfr. Figure 5).

According to [52,53], the ion exchange properties of LDHs are the main factors determining their effectiveness in corrosion protection.

We can therefore condense what has been said so far by pointing out that despite the widespread use of traditional corrosion protection methods, such as sacrificial anodes, galvanization, and barrier coatings, their performance is often hindered by intrinsic limitations: short lifespan, poor adaptability to dynamic or aggressive environments, and high maintenance costs. These drawbacks underscore the pressing need for smarter and more durable solutions that actively respond to corrosive stimuli [34,35,39].

In this context, Layered Double Hydroxides (LDHs) have emerged as a promising class of 2D nanostructured materials offering both passive and active corrosion protection capabilities. Their distinctive lamellar architecture provides a physical labyrinth barrier, while their high anion exchange capacity enables the capture of aggressive species like chlorides and the controlled release of inhibitors [48,49,50,51,52,53,54,55,56]. These synergistic properties position LDHs as effective self-healing agents within hybrid protective systems.

The transition from laboratory-scale studies to real-world implementation will be briefly considered, even if these topics require further investigation into the scalability, reproducibility, and long-term stability of LDH-based coatings. To date, comprehensive studies on their industrial applicability and cost–benefit ratio remain limited, and these aspects are outside the scope of the present work.

Therefore, this review aims to provide a critical overview of the underlined properties of LDHs, giving examples of recent developments in LDH-reinforced organic coatings. First, the structure and methodologies of synthesis will be presented. Next, we will discuss corrosion protection modes such as (i) hydrophobic and self-repairing properties, (ii) physical barrier formation, and (iii) chloride trapping capacity. The main mechanisms of anticorrosion in composite systems will also be examined, and the authors will identify key challenges and outline future directions to enable the practical application of LDHs in demanding industrial environments [54,55,56].

## 2. Structure and Properties

**Figure 6:**
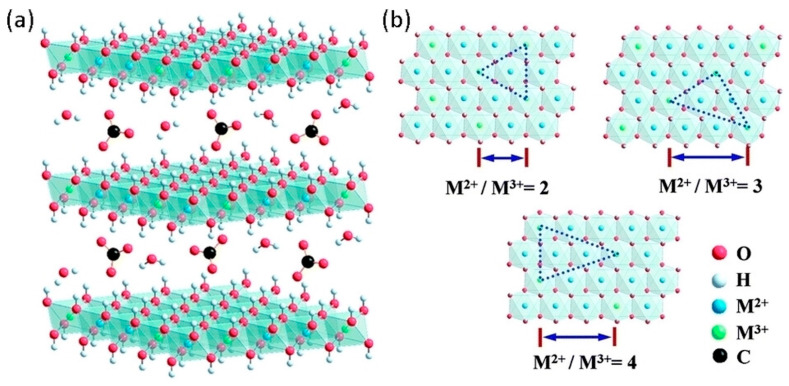
(**a**) General structure of LDHs with idealized interlayer anion as carbonate; (**b**) crystal structure of LDH with different “x” values. Reproduced with permission from Chem. Electro Chem. published by John Wiley and Sons, Creative Commons CC BY license [59].

LDHs, including hydrotalcite and structurally related compounds, are a class of three-dimensional crystalline materials characterised by the ordered arrangement of two-dimensional octahedral layers at the nanoscale [57]. The crystal structure of LDHs is closely related to their physical and chemical properties. A thorough understanding of the crystal structure is crucial for the optimisation of the material’s macroscopic properties. Currently, there are numerous studies on their structure; among the various proposals, the most widely accepted structural form is the following:

$${\left[M_{1-x}^{2+}M_{x}^{3+}{\left(\mathrm{OH}\right)}_{2}\right]}^{x+}{\left[A^{n-}\right]}_{x/n}\cdot {\mathrm{yH}}_{2}O$$

where M^2+^ and M^3+^ represent bivalent (Mg^2+^, Zn^2+^, Ni^2+^, Co^2+^, etc.) and trivalent (Al^3+^, Co^3+^, Fe^3+^, Mn^3+^, etc.) metal cations in the main layers; A^n−^ indicates organic or inorganic anions located in interlamellar spaces, such as ${\mathrm{CO}}_{3}^{2-},{\mathrm{SO}}_{4}^{2-},{\mathrm{Cl}}^{-},{\mathrm{NO}}_{3}^{2-}$, mercaptobenzothiazole anions, and organic phthalates; x is the molar ratio M^3+^/(M^2+^ + M^3+^), typically between 0.20 and 0.33; y represents the number of water molecules contained between the layers [58]. Structurally, part of the bivalent cations (M^2+^) is replaced by trivalent cations (M^3+^) with similar ionic radii, generating a net positive charge in the lamellar layers. This charge is neutralised by anions located in the interlamellar spaces, giving overall electrical neutrality to the material. The general model of such a structure is shown in Figure 6 [59].

The metal cations occupy the centre of each octahedron and are coordinated to six hydroxyl groups (OH^−^), forming the main hydroxyl lattice. The interlamellar anions are bound to the lattice via electrostatic interactions, hydrogen bonds, and other weak forces. During the synthesis process, water molecules fit between the layers, further stabilising the structure [60,61,62,63].

Due to the aforementioned anion exchange capacity, anion-type corrosion inhibitors can be effectively intercalated into the interlayer spaces of LDHs, imparting protective properties to metals and their alloys.

**Figure 7:**
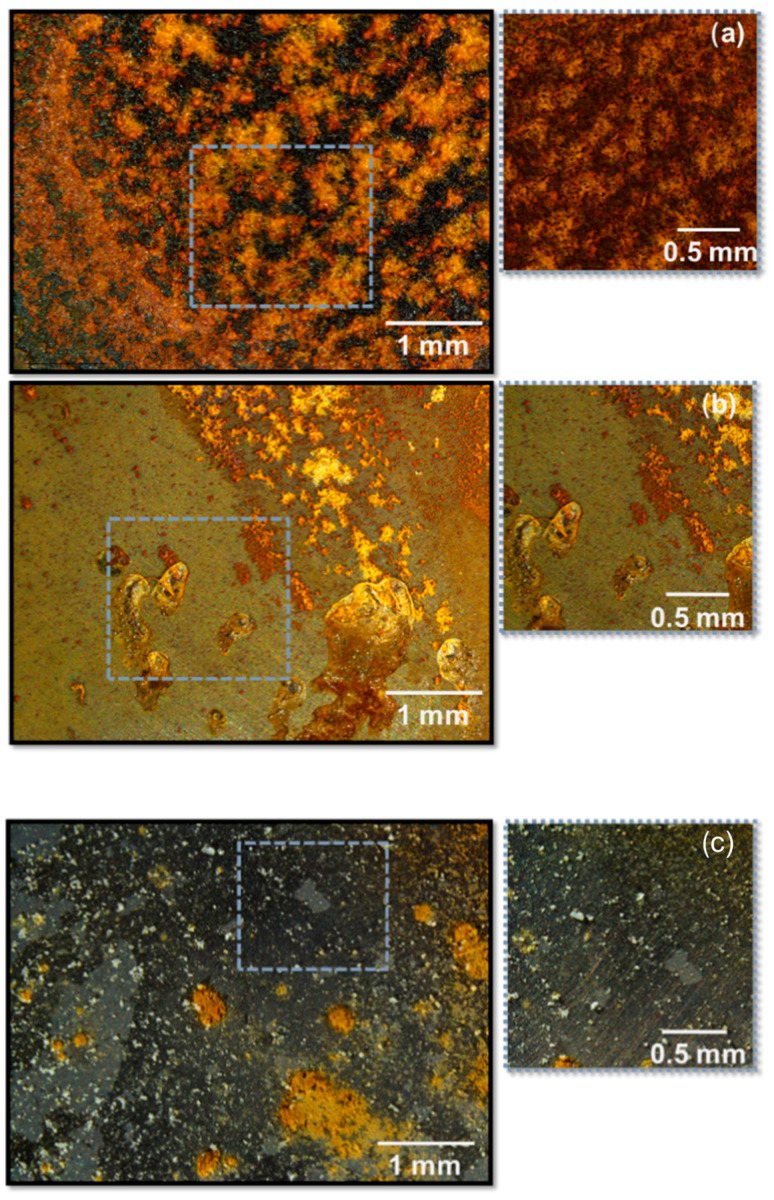
Cast iron substrates after 1-week immersion (**a**) in a 50 mM NaCl solution, (**b**) in a 50 mM NaCl + 5 mM Na_2_HPO_4_ solution, and (**c**) with addition of 5 mM Mg(2)Al-H_2_PO_4_ LDH. Reproduced with permission from Surface & Coating Technology, published by Elsevier, 2019 [58].

For example, Figure 7 clearly shows the effectiveness of Mg-Al LDHs intercalated with corrosion inhibitive dihydrogen phosphate anions in protecting cast iron immersed in a 50 mM NaCl + 5 mM Na_2_HPO_4_ solution [58]. After the same period of immersion, the sample with LDHs (b) shows less pitting corrosion than the one without LDHs (a), although the amounts of phosphates in both tests were the same. In fact, LDHs act as nano-containers of inhibitive species; they release H_2_PO_4_ and capture Cl^−^.

**Figure 8:**
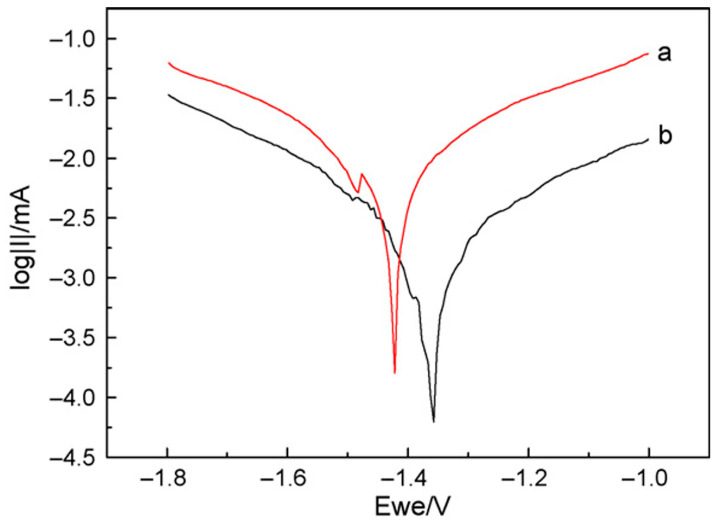
Polarization curves of the bare AZ31 substrate without (a) and with (b) LDH film. Reproduced with permission from the J. Alloys Compd., published by Elsevier, 2010 [64].

Another typical example of the improvement of corrosion resistance provided by LDHs is given in the work of Wang et al. [64]. An Mg–Al LDH film was grown on the AZ31 alloy through the hydrothermal crystallization method. Samples with and without the coating were then immersed in a 3.5% NaCl aqueous solution. To highlight the effect of LDH film, Figure 8 compares the polarization curves obtained in potentiodynamic polarization tests of the samples. In a polarization curve, lower corrosion current density corresponds to lower corrosion rate and better corrosion resistance; therefore, the results suggest that the LDH film can provide effective corrosion resistance.

In what follows, a summary of the role of LDHs in the corrosion protection of metal substrates is provided. Both the corrosion inhibition strategy based on the microstructural characteristics of LDHs and the main factors influencing the anticorrosion effectiveness are described, with reference to the composition and release dynamics of the intercalated inhibitors.

## 3. Synthesis Procedures of LDHs

After years of study, the use of LDHs in the corrosion protection of metals has recently undergone considerable progress. Currently, numerous coatings of LDHs have been developed through different synthesis strategies, based on mixing metal salts or oxides with alkaline solutions. These strategies can be divided into two main macro-categories:

- **Direct Methods**: LDHs are directly synthesized onto the substrate, in the form of well-organized, continuous, and uniform layers. Those layers play the role of Conversional Coatings (CCs) that can be employed as synthesized or post-treated. In this last case, they can preserve their structure (as the cases of polymer–LDH hybrid coatings, superhydrophobic LDH CC, LDH CC functionalized via ion exchange, etc.), or they can be transformed (through thermal treatment harnessing the memory effect or by controllable recrystallization of LDH CC) into Metal Organic Framework Conversional Coatings (MOF CCs).
- **Indirect Methods**: LDHs can be integrated in the form of powders (or pigments) with base material and the resultant coating is applied over the surface. Indirect methods are additional treatments and subsequent modifications of preformed LDHs not yet in the form of CCs.

The main routes linked to these two categories are reported in Table 1.

These methods exploit two key properties of LDHs: the versatility in the choice of metals forming the lamellar layer and the capacity for anion exchange in the interlamellar spaces. Therefore, it is possible to obtain nanostructures with diversified morphologies like powders or thin films in cationic, anionic, or multi-metal doped forms, depending on the method adopted and the composition chosen. In what follows, we will limit ourselves to a brief description of the most used growth techniques for anti-corrosive coatings.

One of the most common methods to produce LDHs is **co-precipitation**, which is divided into two main variants: co-precipitation at constant pH (steady-state) and co-precipitation at variable pH (unsteady) [65]. For these methods, LDH precursors are synthesised in powder form.

In the first case, also known as the pH-controlled method [66], two solutions containing metal cations and intercalating anions, respectively, are introduced simultaneously into a reactor under vigorous stirring. Crystallisation takes place at a controlled temperature, followed by centrifugation, washing, and drying of the product to obtain solid LDH particles. The pH of the mixture is maintained between 8 and 9 using alkaline solutions (NaOH or Na_2_CO_3_), the optimal range for the solubilisation of most metal salts [67]. In the variable pH method, on the other hand, the solution containing the metal cations is gradually added to an alkaline solution that already contains the desired anions while maintaining constant stirring. During the reaction, the pH is not kept stable but varies continuously [68].

An effective alternative is the **hydrothermal method** [69,70], in which metal oxides or hydroxides are mixed with an alkaline solution, placed in a closed reactor at temperatures between 120 °C and 180 °C and high pressures, for a defined period, promoting nucleation and controlled lamellar growth. The hydrothermal conditions ensure high crystallinity [71,72]. Other advantages are operational simplicity, low environmental impact, the formation of regular and morphologically well-defined structures, and direct applicability on substrates. Both co-precipitation and hydrothermal methods produce LDHs in the form of powders.

The **in situ growth method** consists of directly treating a substrate under specific conditions, so that the LDH film is formed directly on its surface, without first synthesising the material in powder form. The coatings thus obtained are compact, uniform, and generally independent of the geometry of the substrate. In this respect, it is worth mentioning the pioneering work of Leggat, Zhang, and Buchheit [73,74], which developed hydrotalcites as conversion coatings to protect metallic materials.

When the substrate is a metal or alloy, it actively participates in the film-forming process, improving the adhesion between the coating and the base material, making it resistant to detachment. This makes in situ growth one of the most promising strategies for making films of LDHs on metal surfaces.

Both in situ growth and the hydrothermal method allow the production of LDH coatings with varying compositions and anion types, independently of the substrate. For example, Zhang et al. [75] proposed a new approach to obtain ternary LDH coatings doped with cerium (Ce) on aluminium alloys using a urea hydrolysis reaction, and the coating demonstrated excellent anti-corrosive properties. Chen et al. [48] initially applied a micro-arc oxidation (MAO) pre-treatment to the AZ31 magnesium alloy to generate a porous oxide layer enriched with aluminium and magnesium species. Subsequently, a graphene oxide (GO)/MgAl–layered double hydroxide (LDH) composite coating was synthesized via hydrothermal in situ transformation. This process utilized a pure GO aqueous medium, wherein the aluminium and magnesium oxides embedded in the MAO layer acted as intrinsic metal ion sources for LDH nucleation and growth, thus eliminating the need for external salt precursors.

Zhao et al. [76] applied the in situ steam coating technique for the deposition of Mg-Al-based layered double hydroxide (LDH) films (Mg-Al-CO_3_-LDH) on AZ31 and AM30 alloys, using an aqueous solution containing NaOH.

The steam coating, also known as steam-assisted coating, is a simple and environmentally friendly hydrothermal methodology that enables the growth of LDH films on metal substrates (e.g., magnesium, aluminium, zinc, or iron alloys) by exclusively utilising high-temperature water vapour without the need to introduce exogenous metal salts. This is made possible by the mobilisation of endogenous metal ions present in the substrate, which react with atmospheric anions to generate the protective film.

In contrast to conventional hydrothermal synthesis, which employs aqueous solutions containing metal precursors and generates the films by heterogeneous precipitation, in steam coating, the fluid phase consists exclusively of water vapour, and film formation occurs through controlled oxidation of the base metal.

**Figure 9:**
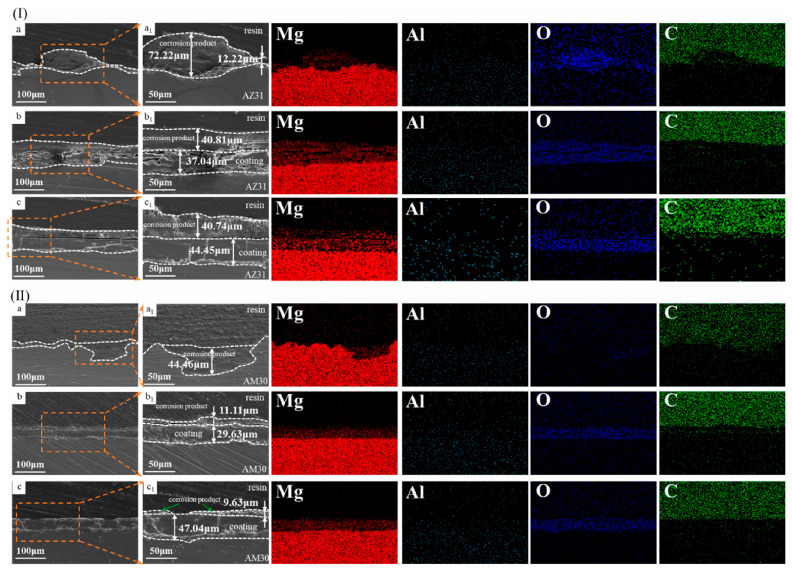
SEM images of longitudinal sections and corresponding elemental distributions of different samples of (**I**) AZ31 and (**II**) AM30 alloys: (**a**) substrate, (**b**) X-NaOH-0, and (**c**) X-NaOH-0.01, after 42-day salt spray experiments and corresponding local magnification images. X stands for AZ31 or AM30. Reproduced with permission from Smart Materials in Manufacturing, published by Elsevier, 2021 [76].

In their study, the authors observed that the presence of NaOH at a concentration of 0.01 M in the reaction medium significantly favoured both the morphology and thickness of the formed LDH film. In particular, the AM30 sample treated with 0.01 M NaOH showed the most compact and uniform surface as well as the highest coating thickness. Furthermore, the corrosion current density of the treated samples was reduced by three orders of magnitude compared to the respective bare substrates. This improvement was attributed to the increased pH induced by the presence of NaOH, which promotes the selective dissolution of the aluminium phase in the substrate and, consequently, accelerates LDH film growth (cfr. Figure 9).

**Figure 10:**
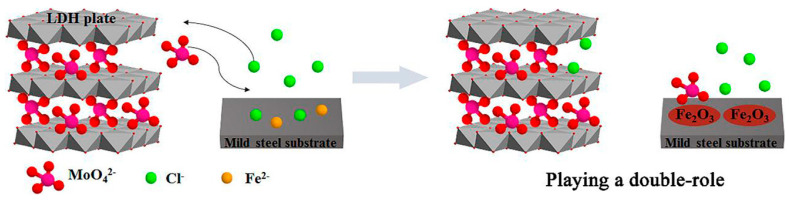
Schematic representation of anions entrapped within LDH framework. Reproduced with permission from Colloid and Interface Science Communications, published by Elsevier, 2018 [78].

As widely emphasised, the structure gives LDHs a marked **anionic exchange** capacity, which is exploited for anti-corrosion protection [77]. As shown in Figure 10, the anion exchange involves (i) diffusion of the anion from the external medium towards the interlamellar spaces of the LDH; (ii) removal of the original intercalated anion and replacement with the new anion, driven by electrostatic interactions and hydrogen bonds; (iii) structural readjustment of the LDH layers to accommodate the new anion, with possible variation of the interlayer distance and rearrangement of water molecules [78].

The effectiveness of the exchange depends on several factors, including cationic composition of the LDH layer (M^2+^/M^3+^), which regulates the charge density and spatiality of the interlamellar layer [77] and chemical–physical properties of the anion: ionic radius, charge density, hydration, and chemical affinity. Anions with high charge density (e.g., CO_3_^2−^) show strong affinity and are difficult to exchange, whereas anions such as NO_3_^−^ o Cl^−^ are more easily exchangeable [79]. Environmental conditions, i.e., pH, temperature, anion concentration and reaction time, also influence the kinetics and equilibrium of the exchange [80].

The process can be described by considering three mechanisms:

- ***Direct exchange***—The direct mechanism involves the exchange of anions present between the lamellar layers of LDHs (e.g., NO_3_^−^ o Cl^−^) with anions present in the reaction medium, driven by electrostatic interactions and hydrogen bonds with the OH-M^2+^/M^3+^ layer. The initial stage can lead to a partial expansion of the lattice, facilitating anion transfer. Molecular Dynamic (MD) simulations show that anion substitution is controlled by both the ionic size and the affinity for the lamellar layers [81].
- ***Exchange via acid attack***—In an acidic environment, the OH^−^ groups of the LDH structure are protonated and the lamellar network partially dissolves, releasing the intercalated anions (e.g., CO_3_^2−^). This controlled dissolution opens up space for the entry of new anions (e.g., Cl^−^ o NO_3_^−^) once the environment returns to a less acidic pH [82]. This approach allows the exchange of “strong” anions, exploiting structural instability under acidic conditions.
- ***Exchange by formation of surfactant salts***—In this method, a surfactant anion (e.g., dodecyl sulphate, DBS) is intercalated into the interlamellar spaces, significantly changing the interlayer spacing. Subsequently, when interacting with a cationic surfactant (e.g., cetyltrimethylammonium bromide, CTAB), an organic salt is generated that “exits the lattice”, leading to a forced expulsion (“squeeze-out” mechanism) of the original anion and opening space for the intercalation of new anions [83,84]. This allows the formation of lamellar films with a porous structure and selective encapsulation capability.

A brief comparison of the three mechanisms is given in Table 2.

Regarding **non-anionic exchange**, a distinction must be made between the Delamination-Filling mechanism and the Reconstruction mechanism. More precisely:

- ***Delamination-Filling Mechanism* (Exfoliation-Assembly)**

The Delamination-Filling process is based on the separation of LDHs into thin nanosheets (1–2 nm), obtained by treatment in polar solvents (e.g., formamide, ethanol) and/or the use of ultrasound. This delamination reduces electrostatic and Van der Waals forces between the layers, leading to a stable dispersion of the lamellae [48,85]. Subsequently, these nanosheets can be re-aggregated into lamellar or composite structures mixed in a controlled manner with anions or functionalising agents, generating hybrid materials or coatings with high surface areas [86]. This strategy yields materials with high surface accessibility, adjustable porosity, and potential application in catalysis, energy storage, and anticorrosive coatings. However, it has limitations in terms of scalability, high solvent usage, and the need for precise operating conditions [48,87].

- ***Reconstruction Mechanism***

**Figure 11:**
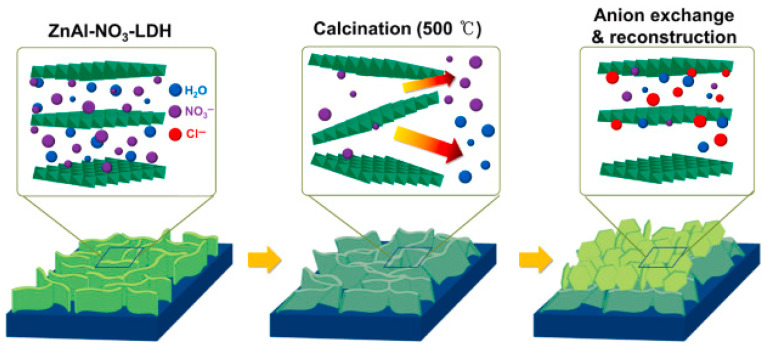
Schematic illustration of the evolution of chemical composition and morphology of ZnAl-LDHs during the reconstruction process. Reproduced with permission from Ceramics International, published by Elsevier, 2022 [88].

Reconstruction is based on the memory effect of Layered Double Oxides (LDOs) obtained by calcination of LDHs. When the LDOs are put in solutions containing anions (e.g., CO_3_^2−^, NO_3_^−^, PO_4_^3−^), they regenerate the original lamellar structure of the LDHs by layer reorganisation and intercalation of the anions [88,89]. Regeneration occurs rapidly (within 5–30 min) even at room temperature and is influenced by factors such as the chemical composition of the precursor, the pH of the medium, the temperature, and the nature of the anion introduced. Reconstruction is advantageous in terms of the simplicity and reversibility of the process but may result in a partial loss of the original crystalline order (cfr. Figure 11) [6,88].

**Figure 12:**
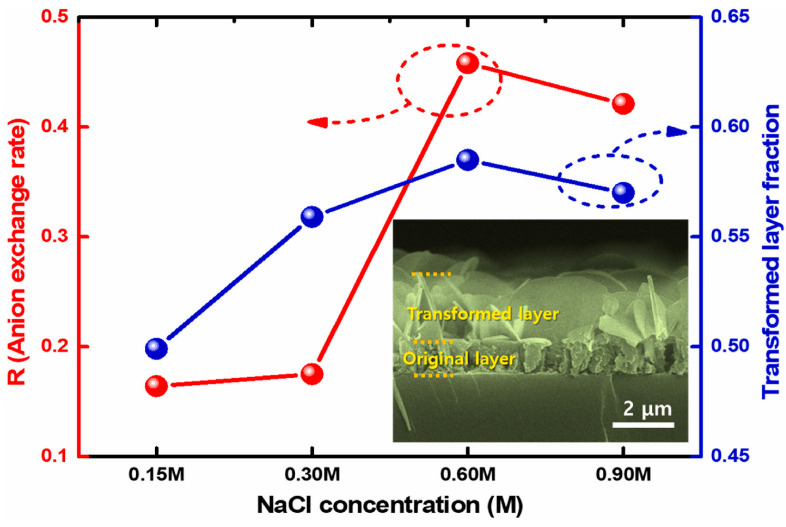
Variation of the anion exchange rate and transformed layer fraction of ZnAl-LDHs with the variation of the NaCl concentration. The inset shows a cross-sectional image of ZnAl-LDH rehydrated in a 0.60 M NaCl solution, clearly showing the interface. Reproduced with permission from Ceramics International, published by Elsevier, 2022 [88].

The speed of the process and the quality of the regenerated material depend on the original composition (M^2+^/M^3+^ ratio), anion concentration, and temperature (Figure 12).

A comparison of the two methods is shown in Table 3.

These mechanisms enable the design of advanced LDH materials with high functional customization, thanks to the possibility of manipulating structure, composition, and interlamellar interactions at the nanoscopic level.

## 4. Corrosion Protection Mechanism

When LDHs are used as corrosion protection for metals and alloys, their effectiveness is limited to neutral or alkaline environmental conditions. This limitation arises from the hydroxyl nature (base) of the crystalline structure of LDHs, which makes them chemically unstable and susceptible to dissolution in acidic environments. Therefore, they are not used directly as corrosion inhibitors, but rather as storage systems for inhibitors. Thanks to their balanced charge structure and ion exchange capacity, they can intercalate a wide variety of “guests”, both inorganic and organic.

The synthesis methods reported in the previous section, possibly combined with surface treatments such as MAO (Micro-Arc Oxidation) and PEO (Plasma Electrolytic Oxidation), have been widely tested to create different morphologies of LDH nanocontainers with cationic, anionic, or double-doped characteristics, depending on the adopted synthesis conditions.

**Figure 13:**
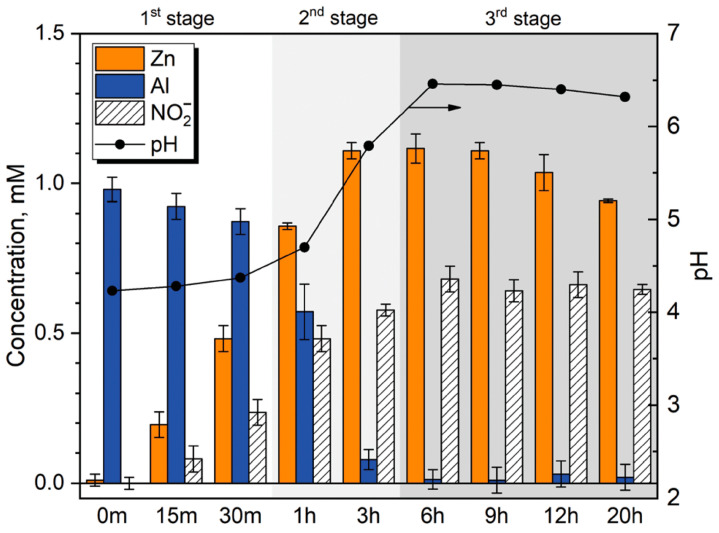
Time-resolved evolution of Al, Zn, and NO_2_^−^ concentrations in solution and their pH (measured at 25 °C) after different times of synthesis. Reproduced with permission from Chemical Communication, published by Royal Society of Chemistry, 2019 [90].

**Figure 14:**
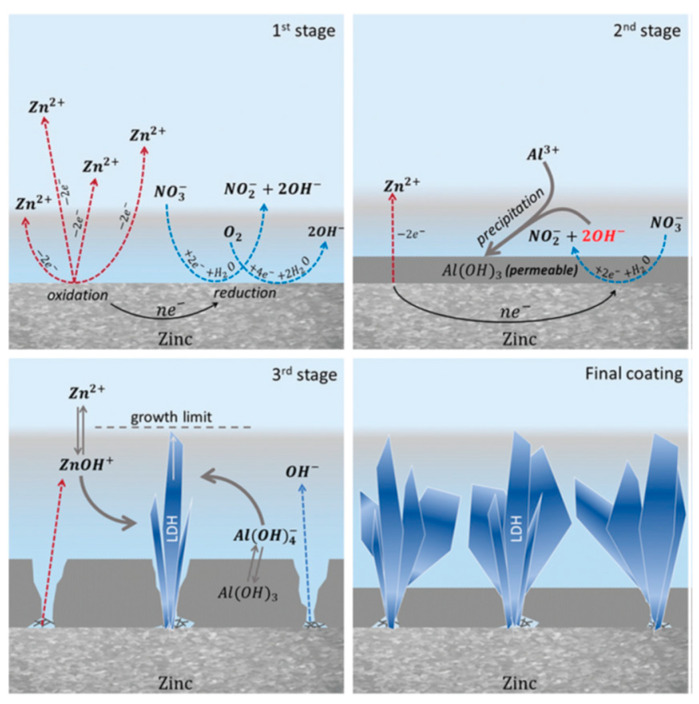
The scheme of the proposed mechanism of LDH growth on zinc in solution of 0.1 M NaNO_3_ + 1 mM Al(NO_3_)_3_. Reproduced with permission from Chemical Communication, published by Royal Society of Chemistry, 2019 [90].

Several studies have highlighted the high affinity of LDHs for carbonate anions, which are easily intercalated but difficult to replace through ion exchange processes [90]. On the contrary, anions such as nitrates and halides are not only easy to intercalate but also easily replaceable (cfr. Figure 13 and Figure 14). Consequently, LDHs intercalated with nitrates are commonly used as precursors for the subsequent incorporation of more complex anions through exchange reactions. LDHs containing carbonate anions, on the other hand, are usually used as reference materials (blanks) for experimental comparisons.

The intercalation capacity of anions in the interlamellar spaces of LDHs follows a well-defined sequence related to valence, charge density, and ionic radius of the anions involved. As regards anions, two examples of sequences related to the order of ease of intercalation are as follows: (i) monovalent anions OH^−^ > F^−^ > Cl^−^ > Br^−^ > NO_3_^−^ > I^−^; (ii) bivalent anions CO_3_^2−^ > MoO_4_^2−^ > SO_4_^2−^ [91]. The affinity of anions towards the LDH matrix follows the following increasing trend: I^−^ < NO_3_^−^ < Br^−^ < Cl^−^ < HPO_4_^2−^ < F^−^ < OH^−^ < SO_4_^2−^ < C_10_H_4_N_2_O_8_S_2_^2−^ < CO_3_^2−^. This implies that the anions located on the left in the sequence can be more easily replaced by those on the right through interlamellar ion exchange processes, but not vice versa.

In response to external stimuli, such as Cl^−^ or SO_4_^2−^, inhibitory ions are released if they have a lower stability than those of, respectively, Cl^−^ or SO_4_^2−^ and the latter, in turn, are simultaneously sequestered by the LDH matrix [92]. Furthermore, the instability in acidic environments confers to LDHs “pH-responsive valve” behaviour, allowing a modulation of the inhibitor release as a function of the nanosheet dissolution rate.

Compared to spherical or tubular structures, these structures present a high length/diameter ratio and offer excellent barrier properties. Possessing numerous hydroxyl groups on the surface of the layers, they allow “surface functionalization” through electrostatic interactions, hydrogen bonds, or covalent bonds with –OH groups. This facilitates the realization of “superhydrophobic” coatings or surfaces infused with lubricating liquids, further improving the physical protection against corrosive agents.

Unlike other functionalized vessels, such as polymeric microcapsules or inorganic materials (e.g., mesoporous silica, zeolites), which require complex surface modifications to respond to environmental stimuli (pH, UV, etc.), it is evident that LDHs offer an intrinsically responsive approach that is easier to integrate into advanced anticorrosion systems.

### 4.1. Cationic Storage

In the literature, some remarkable works describe how LDHs have been employed as cationic nano-vessels. For example, Das et al. [80] obtained MgAlCe-based LDHs by co-precipitation at a constant pH, using carbonate as the balancing anion, through the mixing of Mg^2+^, Al^3+^, and Ce^3+^ nitrates with a solution of Na_2_CO_3_ and NaOH. Similarly, Liu et al. [93] synthesized ZnAlCe LDHs nanoparticles, characterized by plate-like nanosheets. However, co-precipitation has some limitations: it is a time-consuming process, and the obtained materials often show low crystallinity and poor adhesion to substrates. For this reason, it is a widely used technique to produce LDHs in the form of powder, but it is less suitable for the deposition of thin films [50].

Yang et al. [66], through stationary co-precipitation, demonstrated the effectiveness of an inhibition system based on Modified Hydrotalcites (MHTs) intercalated with inorganic inhibitors—in particular, nitrites—well known for their excellent anticorrosive performance in reinforced concrete systems.

**Figure 15:**
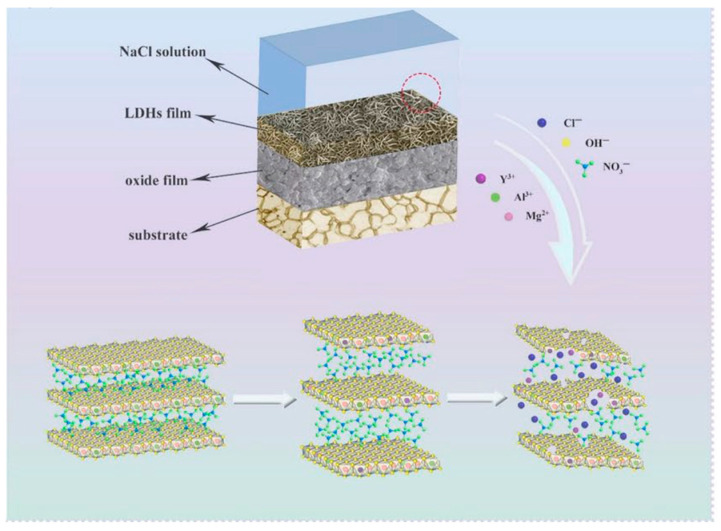
Growth mechanism and electrochemical behaviour of MgAlY-LDHs. Reproduced with permission from Applied Surface Science, published by Elsevier, 2021 [94].

Dai et al. [94] fabricated yttrium-doped MgAl-LDH films on the surface of anodized AZ31 and Mg-Y alloys by hydrothermal synthesis. The incorporation of Y^3+^ ions into the lamellar structure significantly improved the corrosion resistance, also providing the coating with self-healing properties (Figure 15).

Both in situ growth and hydrothermal methods allow producing LDH coatings with variable anion compositions and types, independently of the substrate.

### 4.2. Anionic Storage

The main method for obtaining LDHs intercalated with anionic-type corrosion inhibitors is to exploit the anion exchange properties inherent in the lamellar structure of the materials. Specifically, intercalation occurs by treating the precursor material with a solution containing the desired anion, usually in the form of a soluble salt. The interaction between the positive surface charges of the layers and the target anions promotes the substitution of the original interlayer anions, leading to the formation of the intercalated compound.

**Figure 16:**
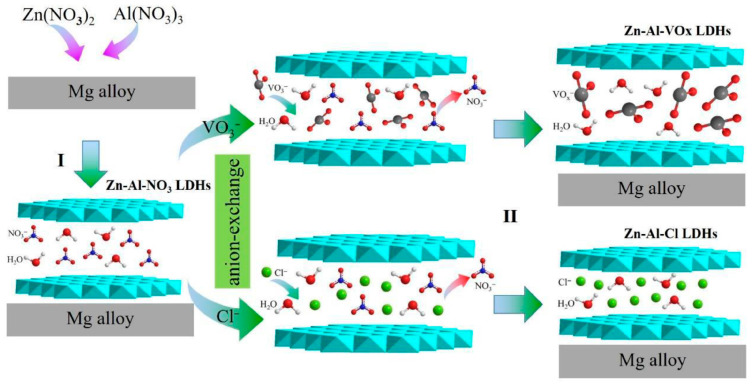
Schematic illustration of formation process of ZnAl-VO_x_ LDH and ZnAl-Cl LDH films on magnesium alloy. Reproduced with permission from Applied Surface Science, published by Elsevier, 2017 [95].

Among the first investigations on this matter, Zhou et al. [95] developed films of Zn-Al LDHs intercalated with NO_3_^−^ on magnesium alloy substrates by hydrothermal synthesis. Subsequently, to improve corrosion resistance, the NO_3_^−^ anions were replaced with Cl^−^ and VO_3_^−^ through an anion exchange process (see Figure 16).

Jiang et al. [96,97] produced a MgAl-LDH-based coating on magnesium alloy substrates by means of Micro-Arc Oxidation (MAO), followed by controlled growth of the LDH film by co-precipitation and hydrothermal treatment. Specifically, a liquid-infused porous surface based on the LDH film was developed, capable of retaining lubricating fluids and inhibitory anions such as MoO_4_^2−^, while effectively sealing defects in the MAO coating. Contact angle measurements showed that the chemically modified coating exhibits superhydrophobicity and super-lubricity properties. EDS analysis conducted before and after ion exchange showed a significant reduction of the element Mo after immersion, while a peak relative to Cl^−^ was detected, suggesting that MoO_4_^2−^ was released from the LDH film in the presence of chloride ions. Furthermore, the partial retention of Cl^−^ in the interlamellar layers confirms the effectiveness of the active inhibitor release mechanism, contributing to the corrosion protection of the metal substrate.

Su et al. [98] obtained MgAl-LDH nanocarriers intercalated with NO_2_^−^ by a method based on oscillation in an acidic environment followed by anion exchange. The obtained nanocarriers were subsequently incorporated into polymer matrices to evaluate the self-repairing effectiveness of the NO_2_^−^-LDH system. Compared to conventional treatments, the employed acidification did not compromise the structural integrity of the LDHs, which retained a regular hexagonal morphology.

**Figure 17:**
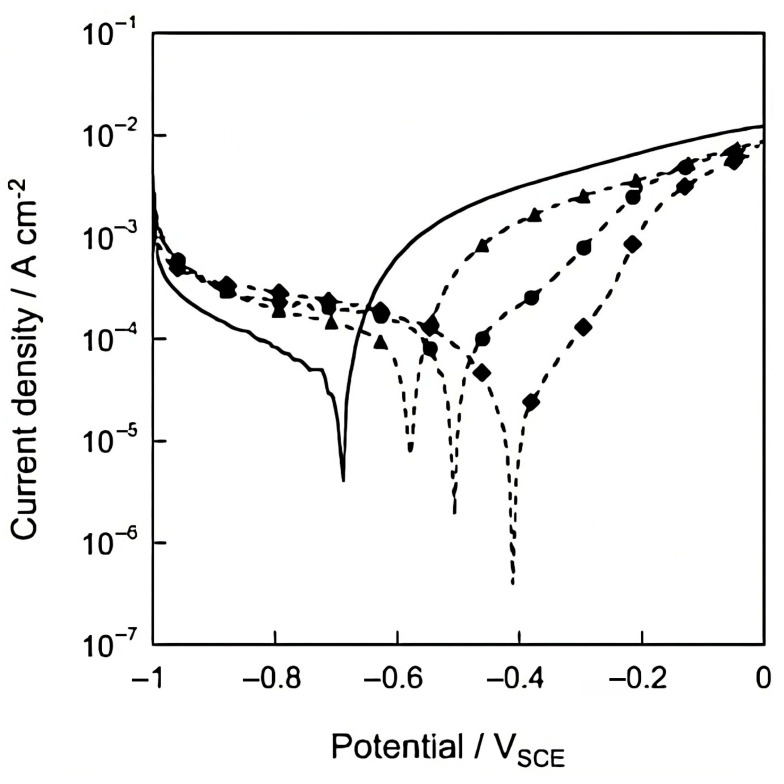
Polarization curves of carbon steel electrode for three LDH–BTSA concentrations after 2 h of immersion in the 0.1 M NaCl solution: (●) 1 g/L; (♦) 3 g/L; (▲) 5 g/L; (-) without inhibitor. Reproduced with permission from Progress in Organic Coatings, published by Elsevier, 2012 [99].

**Figure 18:**
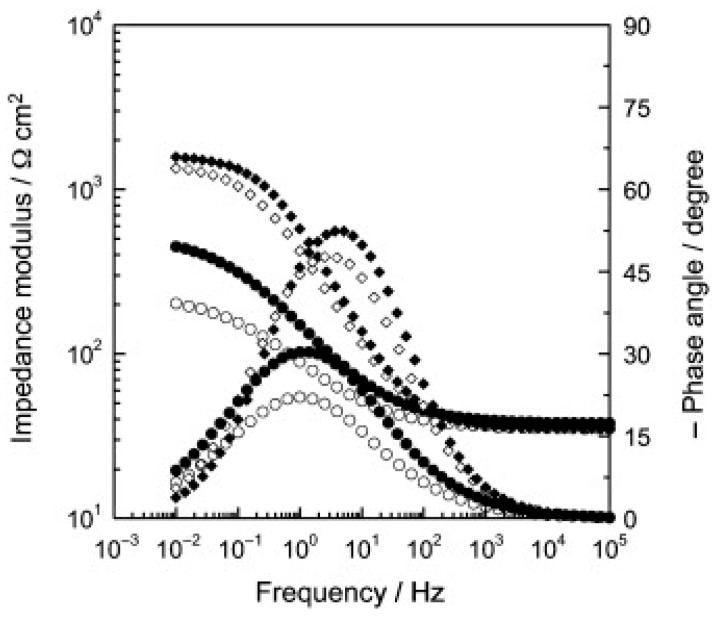
Electrochemical impedance diagrams (Bode representation) obtained for the carbon steel electrode for three LDH–BTSA concentrations after 2 h of immersion in the 0.1 M NaCl solution: (○) without inhibitor; (●) 1 g/L; (♦) 3 g/L; (◊) 5 g/L. Reproduced with permission from Progress in Organic Coatings, published by Elsevier, 2012 [99].

Hang et al. [99] employed 2-benzothiazolylthio-succinic acid (BTSA) as a corrosion inhibitor, intercalating it into MgAl-LDH by co-precipitation. The inhibitor was effectively inserted between the material layers with a loading capacity of about 33%. The release of BTSA was found to be dependent on the NaCl concentration in the electrolyte. The polarisation curves obtained on carbon steel indicated that the LDH-BTSA system acts as an anodic inhibitor, reaching an efficacy of about 90% at a concentration of 3 g/L. Electrochemical impedance analyses also showed that the incorporation of 3% LDH-BTSA into an epoxy matrix significantly improves the corrosion protection of the steel (cfr. Figure 17 and Figure 18).

### 4.3. Chloride Entrapment

It is well known that the penetration and aggressive action of chloride ions (Cl^−^) are decisive factors in the corrosion process of metals. An effective strategy to limit their impact is to prevent the diffusion of Cl^−^ ions within the metal substrate. In this context, LDHs offer a significant advantage due to their ability to exchange anions: they can intercept and retain Cl^−^ ions within the interlamellar spaces, while releasing the anions originally present. This phenomenon, known as the Cl-binding effect or Chloride Entrapment [49], is an advanced mechanism in LDH systems that significantly contributes to corrosion mitigation in chloride-containing environments by reducing the concentration of free Cl^−^ and contributing to the inhibition of the corrosive process [100]. In ZnAl-LDH films intercalated with NO_3_^−^, the trapping of Cl^−^ has been experimentally demonstrated by elemental and spectroscopic analyses [100,101].

**Figure 19:**
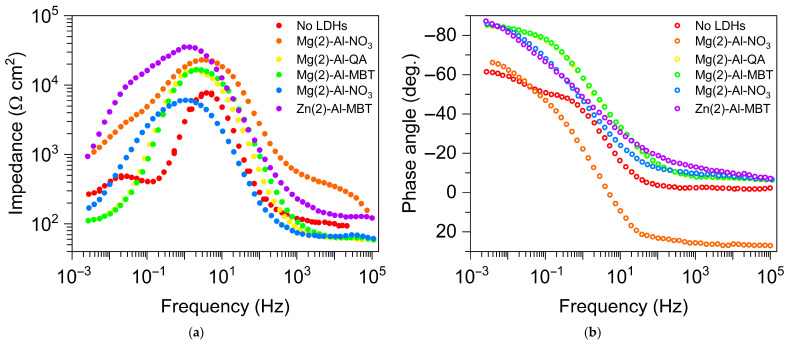
Impedance (**a**) and phase angle (**b**) measured in EIS tests carried out on AA2024 alloy after 7 days in contact with different LDH-containing solutions. Quinaldate (QA), 2-mercaptobenzothiazolate (MBT). Redrawn from ref. [102].

Zn-Al and Mg-Al LDHs, loaded with quinaldate and 2-mercaptobenzothiazolate anions, were synthesized via an anion-exchange reaction by Poznyak et al. [102]. The release of organic anions into the bulk solution is triggered by the presence of Cl^−^; the release of inhibitors and entrapment of aggressive chlorides are sequential processes. The anticorrosion effect of loaded LDHs toward the AA2024 is clearly shown by electrochemical impedance spectroscopy (EIS) measurements: the Bode plots in Figure 19 were obtained after 7 days of contact with different LDH-containing solutions.

The anion exchange process in LDHs preferentially starts along the edges or outer surfaces of the crystals and then progresses within the interlamellar spaces [103]. In this initial stage, there is a rapid replacement of the intercalated anions with aggressive anions, such as Cl^−^, from the corrosive environment. This phenomenon is commonly referred to as the “burst effect” and represents the fastest and most localised exchange stage at the edges of the material [104]. This mechanism involves chloride ions being initially adsorbed onto the outer surfaces of the LDHs, where a rapid exchange occurs with the previously intercalated inhibitory anions. Subsequently, the Cl^−^ ions progressively diffuse within the interlamellar spaces, while the functional anions are released into the solution. The process continues until a dynamic equilibrium is reached, in which the exchange rate between incoming (Cl^−^) and outgoing (inhibitors) anions becomes balanced.

In this configuration, the LDH system acts as an active reservoir: it selectively captures harmful ions such as Cl^−^ and releases corrosion inhibitors, thus reducing the aggressiveness of the electrolytic environment and improving the protection of the metal substrate. The exchange equilibrium is reached when the interlamellar galleries are saturated with Cl^−^ ions and the inhibitor anions are completely transferred into the solution.

It has been observed that under conditions of low chloride ion (Cl^−^) concentration, the predominant mechanism is anion exchange, while at higher concentrations, anion exchange processes and surface adsorption phenomena can coexist [104]. Several parameters influence the dynamics of anion exchange, including the charge density and ionic radius of the anion, concentration of ions in solution, pH, and temperature of the electrolyte [91,99,105].

In a study by Lin et al. [106], magnesium alloys coated with in situ-grown Mg-Al LDH films were immersed in a 0.6 mol/L NaCl solution for 5 h, together with samples without coating. Subsequent elemental analysis showed a significantly higher concentration of Cl^−^ within the LDH layer than the uncoated alloy surface. This result confirms the effectiveness of LDHs in sequestering chloride ions from solution, limiting their interaction with the metal substrate and improving its corrosion resistance.

**Figure 20:**
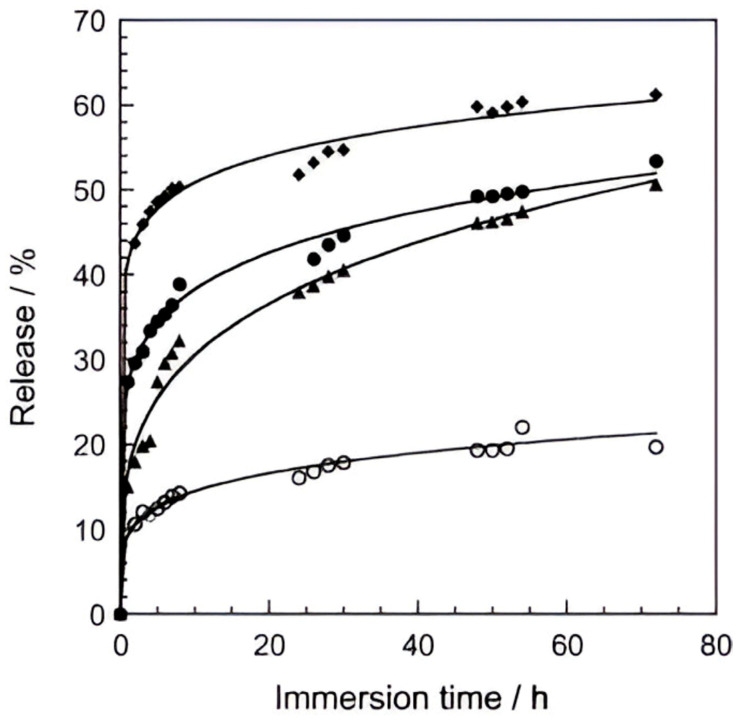
Release curves of BSTA from LDH-BSTA NaCl solution at different concentrations: (○) 0%; (▴) 0.5%; (●) 1%; (♦) 3%. Reproduced with permission from Progress in Organic Coatings, published by Elsevier, 2012 [99].

In addition, increasing the concentration of Cl^−^ in the electrolyte environment promotes greater release of the intercalated inhibitor in LDHs, as it accelerates the rate of anion exchange. This behaviour was confirmed by Hang et al. [99], who studied the release of benzothiazolyl succinate (BTSA) intercalated in Mg-Al LDHs as a corrosion inhibitor for carbon steel, observing a direct correlation between NaCl concentration and the amount of released BTSA (see Figure 20). They also showed that the BTSA anion is released to a greater extent in solutions containing 3 wt % Na_2_SO_4_ than NaCl, attributing the phenomenon to more intense electrostatic interactions between SO_4_^2−^ and positive LDH layers. Multicaric anions also tend to remain better intercalated due to the coulombic bonding effect.

Alkaline conditions favour the selective release of inhibitors from LDHs and modified clay materials [107], suggesting how an increase in local pH, e.g., under neutral corrosion conditions, can activate self-repair mechanisms via controlled release of inhibitory anions. The results showed that ZnAl LDH synthesised at pH 8 had a well-defined structure and the highest inhibitor load. Specifically, 0.1 g/L of ZnAl LDH at pH 8 achieved an impressive inhibition efficiency of 95.18%, as indicated by the potentiodynamic polarisation results.

Structural parameters such as the width of the interlamellar space and the M^2+^/M^3+^ ratio affect the Cl^−^ trapping capacity: a wide interlayer facilitates binding, while a high proportion of bivalent cations reduces efficacy due to a decrease in the net positive charge [108].

The final Cl^−^ trapping effect reduces the aggressiveness of the environment, mitigating corrosion and prolonging the life of the metal substrate. In this way, LDHs perform a dual function, acting as traps for aggressive species and reservoirs of corrosion inhibitors [99,109].

### 4.4. Labyrinth and Physical Barrier Mechanism

**Figure 21:**
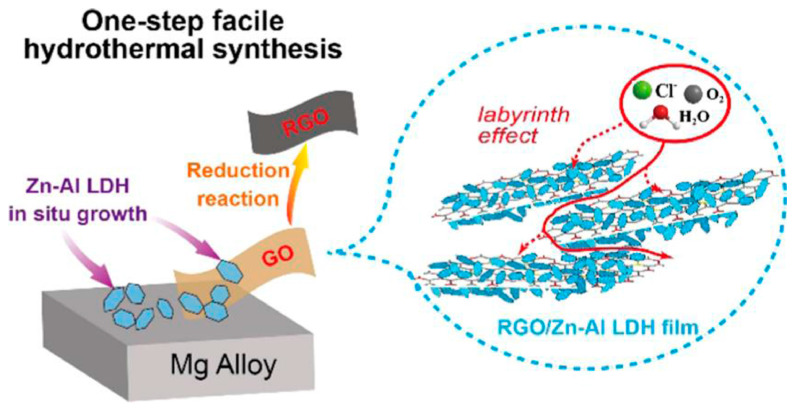
A scheme describing the synthesis process of reduced graphene oxide/layered double hydroxide (RGO/ZnAl-LDH) film and the anticorrosion mechanism. Reproduced with permission from the Journal of Materials Science & Technology, published by Elsevier, 2022 [104].

The integration of functionalised LDH particles within polymer matrices increases the tortuosity of diffusion pathways, limiting the permeation of water, oxygen, and chloride ions. Hybrid coatings consisting of Mg-Al LDH and graphene have shown significantly higher corrosion resistance than unmodified acrylic coatings, due to the synergistic barrier effect created by the combination of LDH and graphene, which prolongs the time required for aggressive species to reach the coating/substrate interface (see Figure 21) [110,111].

In recent studies, LDH films deposited directly on the substrate have demonstrated an effective physical barrier function, preventing direct contact between aggressive species and the metal surface [112,113]. The addition of graphene further improves the structural density of coatings, increasing resistance to the diffusion of corrosive agents and enhancing long-term stability.

In a study of Cao et al. [104], a reduced graphene oxide/zinc–aluminium LDH (RGO/Zn-Al LDH) film was in situ synthesized on a magnesium alloy substrate starting from a solution containing graphene oxide (GO) powder. The RGO/Zn-Al LDH film showed good corrosion resistance with a lower corrosion current density (0.546 μA/cm^2^) than that of the bare substrate (33.2 μA/cm^2^). A schematic view of the synthesis process of reduced graphene oxide/layered double hydroxide (RGO/ZnAl-LDH) film and the anticorrosion mechanism is shown in Figure 21. This method provides a simple and facile approach for enhancing the corrosion protection performance of LDHs.

The physical barrier function is the primary protective mechanism of LDH-reinforced organic coatings. To achieve more effective corrosion protection, the development of multifunctional coatings that integrate advanced shielding capabilities with the passive defence of the substrate is crucial.

### 4.5. Self-Healing Mechanism

LDHs can form stable, insoluble protective films on metal surfaces, helping to mitigate corrosion through their role as a physical barrier and ability to trap aggressive chloride ions. However, under real operating conditions, these films can be mechanically damaged, thus losing their protective effectiveness and even promoting corrosion initiation. To overcome this limitation, LDH-based coatings with self-repairing properties have been developed. When LDHs are loaded with corrosion inhibitors, they function as controlled-release systems that are sensitive to external stimuli. In corrosive environments containing Cl^−^ ions, an ion exchange takes place between the intercalated anions (the inhibitors) and the chlorides in the solution. This process triggers the gradual release of the inhibitors, which migrate towards the exposed metal surface. Once they reach the metal surface, the inhibitors interact with dissolved metal cations (e.g., Mg^2+^, Al^3+^ or Zn^2+^), forming chelates, stable coordinated complexes in which the inhibitor binds to the metal in several places (multidentate function). Chelates are insoluble and thermodynamically stable, forming a new adherent protective film that acts as both a physical and chemical barrier. This mechanism makes it possible not only to prevent corrosion but also to locally repair damage, thus prolonging the effectiveness of the coating over time [34,114].

The anion exchange mechanism plays a crucial role in LDH systems, as it reduces the aggressiveness of the corrosive environment and slows down the local corrosion rate. Global and local electrochemical measurements on LDH systems loaded with inhibitors clearly show this self-repairing ability.

**Figure 22:**
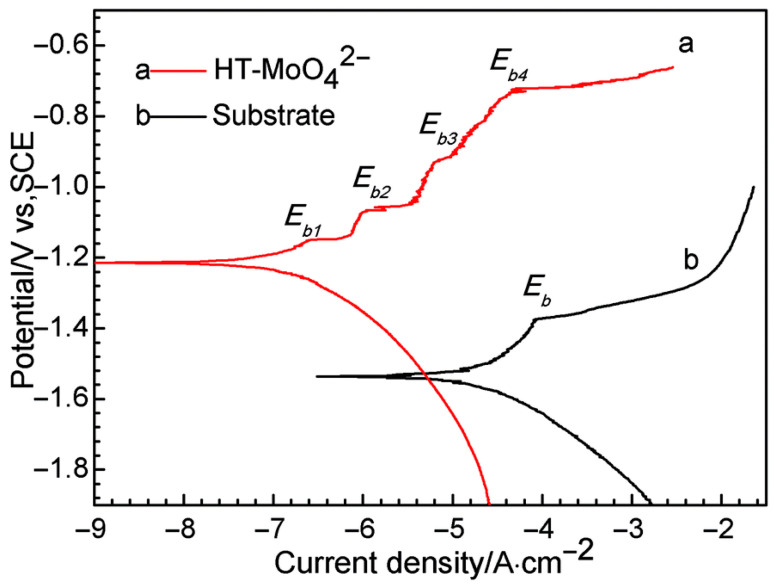
Self-healing of a molybdate-intercalated LDH coating investigated with PPT. Reproduced with permission from the Int. J. Electrochem. Sci., published by Elsevier, 2020 [115].

A case study concerns benzoate intercalated in LDH Zn-Al: on Q235 steel immersed in 3.5 wt.% NaCl, impedance spectroscopy experiments (EIS) show an increase in charge transfer resistance (R_ct_) and a decrease in the Q parameter, indicative of a controlled release and formation of a protective film on the substrate [115]. The decrease in Q reflects a decrease in reactivity at the coating–substrate interface, confirming the self-healing effect of LDHs (cfr. Figure 22).

Inhibitory anion release curves typically show a rapid initial phase followed by a stable plateau [91]. In addition, surface modifications of LDHs can improve both the amount of chargeable inhibitor and its controlled release. For instance, Olya et al. [116] and Wang et al. [117] showed that surface decoration of Zn-Al-Mo_4_^2−^ LDH with SiO_2_ increases the loading capacity and protective efficacy of molybdate.

### 4.6. Hydrophobicity

Hydrophobic characteristics are a crucial aspect in the corrosion protection of organic coatings containing LDHs. These properties significantly contribute to reduce, and in some cases prevent, the penetration and diffusion of corrosive species, thus improving the protective performance of the coating.

The low surface energy of LDHs can be achieved by engineering their composition and interlamellar structure, exploiting the ion exchange mechanism. This gives the coating excellent hydrophobicity, reducing its affinity for water and electrolytes.

Functionalised LDH-based composite coatings with hydrophobic properties thus represent a promising and effective strategy for metal corrosion mitigation due to their dual barrier action and ability to modulate interaction with the aggressive environment.

Intercalated anions in LDHs can impart hydrophobic properties to the structure, a quality that can be induced synthetically during material preparation. Hydrophobicity, enhanced by the chemical nature and morphology of the intercalated anions, contributes significantly to the reduction of electrolyte diffusion through the coating matrix [118,119].

**Figure 23:**
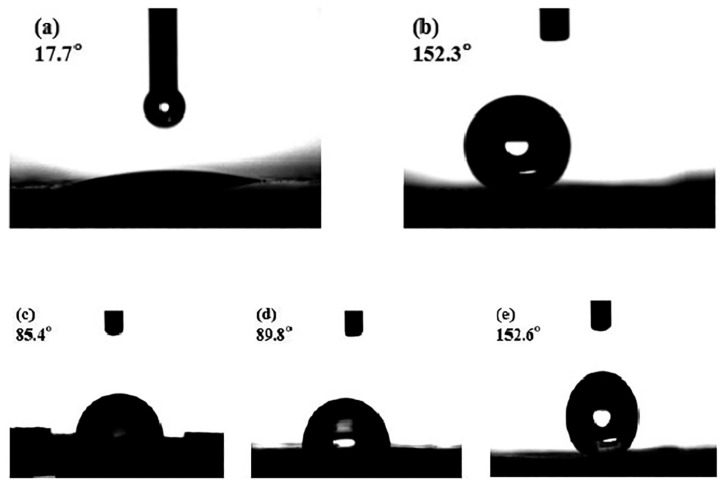
Shapes of water droplets on the surface of different specimens and the corresponding water CAs: (**a**) LDH-F, (**b**) LDH-F-St, (**c**) Pure EP, (**d**) LDH-F-EP, and (**e**) LDH-F-St-EP. Reproduced with permission from Surface and Coatings Technology, published by Elsevier, 2021 [120].

**Figure 24:**
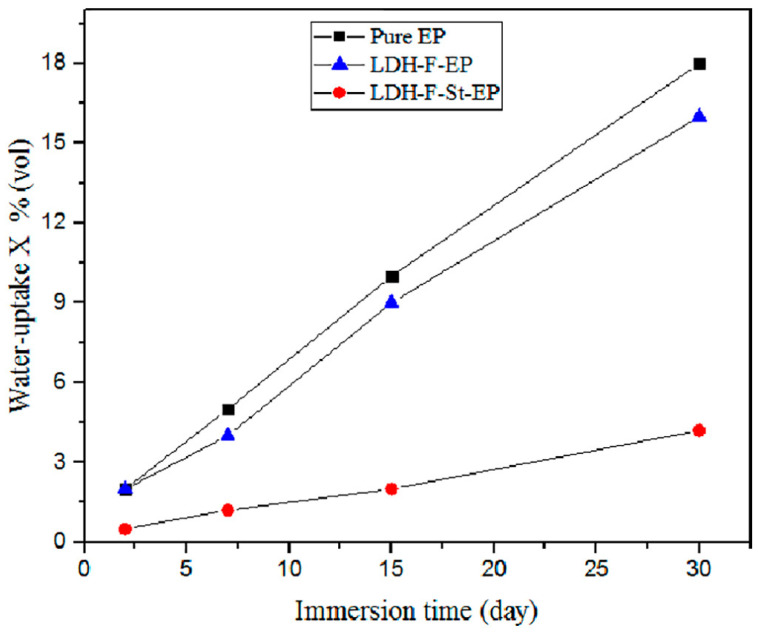
Water uptake (X) for composite coatings during immersion: Pure EP, LDH-F-EP, and LDH-F-St-EP. Reproduced with permission from Surface and Coatings Technology, published by Elsevier, 2021 [120].

Cao et al. [120] developed epoxy coatings with enhanced hydrophobic properties and anti-corrosive performance using MgAl-LDH nanosheets modified with stearate (St) and fluoride (LDH-F-St) anions. The modification with sodium stearate did not alter the morphology and structure of the materials but gave the composite a high hydrophobicity, as demonstrated by the contact angle of more than 150°, indicative of superhydrophobic behaviour (cfr. Figure 23). In other words, when the superhydrophobic surface was immersed in solution, an “air valley” film was formed on the surface, acting as a strong physical barrier against water molecules (cfr. Figure 24) and aggressive ions. Therefore, the corrosion resistance was dramatically improved in this way.

In a further study [52], the same group synthesized a superhydrophobic ZnAl–layered double hydroxide intercalated with laurate and modified with lanthanum (ZnAl-LDH–La), showing successful laurate incorporation confirmed by XRD. The coating exhibited high chemical stability under harsh conditions (temperature, UV, acidic/alkaline media) and provided effective long-term corrosion protection to aluminium in a 3.5 wt.% NaCl solution, indicating strong potential for practical anticorrosion applications.

### 4.7. Substrate Microstructure and Quality of LDH Coating

A relevant issue in corrosion protection is the homogeneity and quality of LDH coating when it is directly grown on the metal surface. As different studies show, this aspect is strongly influenced by microstructural characteristics of the substrate.

**Figure 25:**
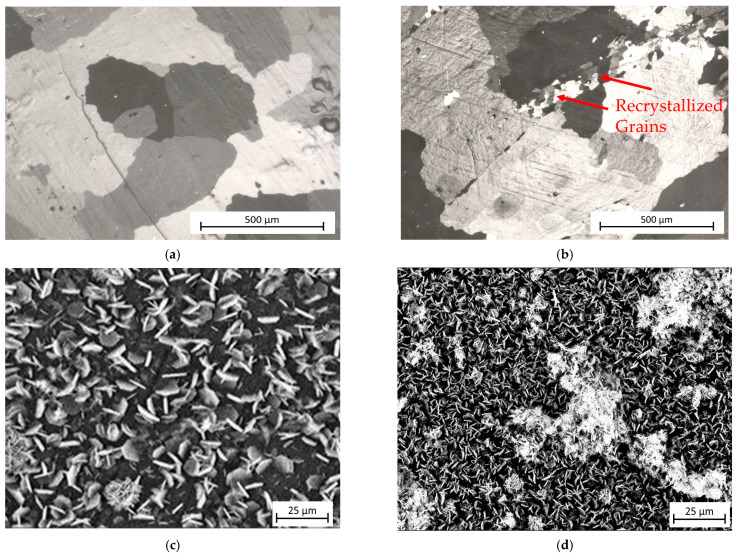
Aluminium substrate with monomodal (**a**) and bimodal (**b**) grain-size distribution. LDHs grown on samples with monomodal (**c**) and bimodal (**d**) grain-size distribution.

Clear evidence comes from experiments of the present authors, who prepared Al-Zn LDHs coatings on substrates of aluminium (purity 99.99%) with monomodal (deformed) and bimodal (deformed and partially recrystallized) grain-size distribution. As shown in Figure 25, the bimodal distribution leads to a non-homogeneous covering and the formation of petal clusters. This is due to two relevant microstructural factors: (i) the presence of two types of grains with quite different sizes; and (ii) the recrystallization of smaller grains, namely those free from dislocations.

Another example is represented by the functionalization of the surface of magnesium alloys by means of LDHs deposition. These materials are of great interest for biomedical applications, specifically in orthopaedics, due to their combination of mechanical and biological properties. Their Young’s modulus (41–45 GPa) is close to that of human bone (3–20 GPa), a characteristic that mitigates the effect of stress shielding [121,122,123,124]. Such alloys also exhibit high biocompatibility and generate non-cytotoxic corrosion products [125] and are biodegradable in body fluids, which makes it possible to avoid second surgical interventions for the removal of implants. Due to these characteristics, AZ31 alloy has recently been used to produce osteosynthesis devices for multiple rib fractures [126,127]. Nevertheless, rapid degradation in physiological environments is a critical limitation, potentially compromising device integrity prior to complete bone regeneration [128,129,130]. Side effects of corrosion include molecular hydrogen formation and local pH elevation, which can lead to tissue necrosis [131,132], as well as marked susceptibility to Stress Corrosion Cracking (SCC) [133,134]. To address these issues, numerous strategies have been adopted [135]. Altering the alloy composition, e.g., by adding rare earths [136,137,138] or metabolic elements such as calcium and zinc [139,140,141], has proven effective. Other solutions include surface treatments (MAO [142], ion implantation [143], HF treatment [144]) and the application of inorganic and hybrid protective coatings [22,145,146,147,148,149,150]. Among the latter, coatings based on LDHs stand out for their combination of high biocompatibility, corrosion resistance, and capacity for the controlled release of therapeutic agents [151,152]. Engineering the microstructure through grain refinement and heat treatment has proven effective in improving mechanical and corrosion resistance [153,154,155,156,157]. The Equal Channel Angular Pressing (ECAP) technique allows for refining the microstructure, increasing the fraction of HAGBs (high-angle grain boundaries), and introduces compressive residual stresses [158,159,160,161,162,163,164].

**Figure 26:**
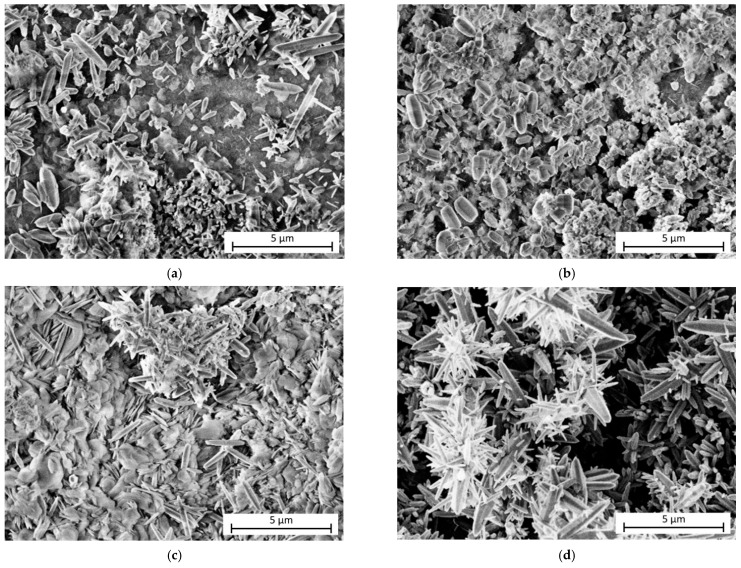
SEM images after LDH synthesis for 30 min: (**a**) 0, (**b**) 1, (**c**) 2, and (**d**) 4 ECAP passes.

The effect of the number of ECAP passes on LDHs grown on AZ31 alloy substrates through in situ co-precipitation and hydrothermal treatment techniques is shown in Figure 26 [165]. LDHs are always present on the surface of the samples; however, the morphology of the coatings varies depending on the number of ECAP passes: the alloy surface subjected to 1 ECAP pass is the only one with a fine, homogeneous, and complete LDH coating (Figure 26b). In the case of 0 passes, the surface covering is incomplete (Figure 26a), while, after 2 (Figure 26c) and 4 passes (Figure 26d), the LDH growth gives rise to clusters of petal-like crystals, which is a not optimal condition for drug release or, more generally, for the LDH interaction with the environment.

These morphological differences depend on LDH formation, which proceeds in two steps, nucleation and growth.

In the first step, screw dislocations emerging at the surface represent the main preferential nucleation sites [166,167]. This dislocation-induced nucleation mechanism is based on the crystalline distortion generated by dislocations, which provides energetically favourable anchorage sites for atoms in solution, based on the fact that atomic diffusion along dislocations (pipe diffusion) is faster than lattice diffusion or diffusion along grain boundaries. Therefore, the distribution of dislocations is a critical parameter for the homogeneity of the coating. A more homogeneous distribution of dislocations, obtained after ECAP processing, leads to a homogeneous surface covering LDH nuclei. This is a pre-requisite to produce a homogeneous covering of LDH crystals; however, it is not sufficient. Then, crystal growth, driven by diffusion, plays a fundamental role because all the nuclei should grow at a similar rate. Otherwise, the preferential growth of some of them will give rise to clusters here and there on the metal surface. Since diffusion is slightly anisotropic in Mg, the grain orientation becomes a critical factor.

The sample subjected to a single ECAP pass has the highest fraction of basal planes parallel to the surface, a condition that maximises the surface diffusion of metal atoms. This effective diffusion leads to homogeneous transport to all areas of the surface, and a continuous and uniform coating is obtained. On the contrary, in the samples subjected to multiple ECAP passes (2 and 4), the unfavourable grain orientation gives rise to uncontrolled growth in localised areas, with the result of petal aggregates, despite the homogeneous nuclei distribution.

**Figure 27:**
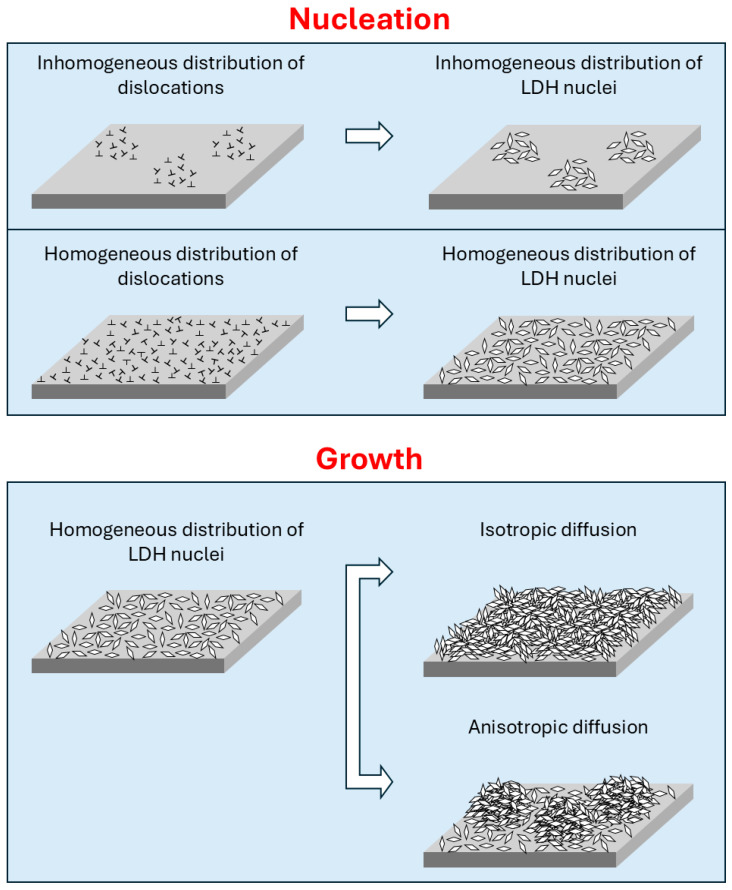
Schematic representation of the role played by substrate microstructural features in nucleation and growth of LDHs.

These concepts are summarized in Figure 27, which shows the role played by substrate microstructural features, especially dislocation distribution and texture, in nucleation and growth of LDHs.

## 5. Commercialization Outlook and Limitations of LDH-Based Coatings

Although numerous studies have demonstrated the effectiveness of LDHs in enhancing anticorrosive performance—through ion exchange, active inhibitor release, and barrier enhancement—their translation into large-scale industrial applications remains limited. Some patents and pre-commercial developments have emerged, particularly in the aerospace and automotive sectors, where LDH-based conversion coatings have been investigated on aluminium alloys such as Al 2024 and Al 7075, serving as Cr (VI)-free alternatives for surface pre-treatment. However, most of these initiatives remain at the laboratory or pilot scale [168].

The primary barriers hindering the commercial deployment of LDH-based coatings on an industrial scale include several interrelated challenges. First, scalability and cost remain critical issues, as LDH synthesis often requires tightly controlled parameters, such as pH, temperature, and ageing time, which are costly and complex to reproduce consistently. Second, stability and compatibility with conventional paint formulations—such as epoxy or polyurethane—can pose dispersion challenges and affect rheology or curing kinetics. Third, despite promising laboratory results, durability under complex, real-world environmental conditions (e.g., marine, aerospace, oil and gas sectors) still lacks long-term field validation. Finally, although LDHs are more environmentally friendly than Cr(VI)-based alternatives, they must undergo rigorous regulatory and standardisation procedures before being approved for critical industrial use.

To bridge the gap between academic research and practical implementation, future studies should prioritise several aspects: (i) demonstrating long-term performance through accelerated ageing tests and real-world exposure trials; (ii) developing cost-effective and scalable synthesis methods, such as continuous co-precipitation or spray deposition; (iii) establishing standardised testing protocols for LDH-based coatings to enable fair comparisons with commercial systems; and (iv) exploring hybrid coating formulations that integrate LDHs with traditional anticorrosive agents to facilitate transitional adoption.

At present, LDHs occupy a niche in the anticorrosion market, with only a limited number of companies actively engaged in R&D and production. Nevertheless, LDHs are already commercially available, produced by both specialised and generalist chemical manufacturers. For instance, Kisuma Chemicals [169] and SMALLMATEK Lda.—a university spin-off from the University of Aveiro (Portugal) [170]—are specialised producers of LDH-based materials for advanced applications. Kisuma Chemicals, the world’s largest producer of synthetic hydrotalcite, offers LDH-based functional fillers, such as the corrosion-resistant pigment DHT-4, used in industries including plastics, pharmaceuticals, agriculture, coatings, adhesives, sealants, and elastomers. Similarly, SMALLMATEK develops LDH-based additives specifically designed for integration into protective coatings aimed at corrosion mitigation on metallic substrates.

In parallel, several multinational chemical companies, such as BASF [171], Clariant [172], Kyowa Chemical Industry Co. [173], and Sakai Chemical Industry [174], supply LDH materials for a broad range of applications, including catalysis, flame retardancy, adsorption, and protective coatings, particularly those tailored to environmental and industrial needs.

## 6. Conclusions

This review has summarised recent advances in the synthesis, morphology control, and anticorrosive performance of Layered Double Hydroxides (LDHs) when applied as protective coatings. LDHs can be either grown in situ on metal surfaces or dispersed within organic matrices, offering self-healing capabilities and long-lasting corrosion resistance in both configurations.

The advancement of LDH-based coatings for corrosion protection relies on four interrelated technical pillars: (i) the selection of metal cations, which determines anion-exchange capacity and inhibitor release efficiency; (ii) substrate-specific behaviour, with excellent performance observed on aluminium and magnesium alloys, but limitations on noble metals and stainless steels; (iii) deposition methods, ranging from incorporation into polymers to electrophoretic and in situ growth techniques; and (iv) control of synthesis conditions, which directly influences LDH morphology and, consequently, coating performance.

Despite their significant potential in industrial and biomedical applications, several critical challenges remain. These include (i) enhancing the understanding of LDH interlayer structure and inhibitor release kinetics, which will enable improved synthesis protocols and the development of materials with controlled morphologies, high crystallinity, and stable, reproducible functional performance; (ii) ensuring chemical compatibility with commercial polymer matrices, which may significantly improve coating density and barrier properties; and (iii) expanding applicability across a wider range of metallic substrates.

Future research should therefore prioritise the development of scalable synthesis routes, long-term field validation, and the design of hybrid, multilayer systems capable of providing intelligent, adaptive, and sustainable protection under aggressive environmental conditions.

## Figures and Tables

**Table 1:** Classification of LDH synthesis techniques.

Direct Methods	Indirect Methods
Hydrothermal Synthesis In Situ Growth Sol-Gel Synthesis Salt–Oxide Reaction Electrochemical Synthesis	Co-Precipitation Anionic Exchange: – Direct – By Acid Attack – By Surfactant Salt Formation Non-Anionic Exchange: – Delamination-Repacking – Reconstruction of LDH

**Table 2:** Comparison of benefits and limitations in different mechanisms.

Mechanism	Benefits	Limitations
**Direct exchange**	Simple, ion selectivity-based	Slow, dependent on affinity and ion size
**Exchange via acid attack**	Enables exchanges with strongly bound anions (e.g., CO_3_^2−^)	Causes structural degradation and requires acidic environment
**Exchange by formation of surfactant salts**	Allows for forced expulsion and specific encapsulation	Requires specific surfactants and organic solvents

**Table 3:** Comparison between Delamination-Filling and Reconstruction methods.

Mechanism	Benefits	Limitations/Technical Challenges
**Delamination-Filling**	High area nanosheet production; flexible reassembly	High cost, use of solvents, difficulty in scaling
**Reconstruction**	Rapid structure reformation; simple	May not fully restore crystalline order

## Data Availability

The raw data supporting the conclusions of this article will be made available by the authors on request.
